# Physical and Behavioral Factors Associated With Improvement in Physical Health and Function Among US Women During Midlife

**DOI:** 10.1001/jamanetworkopen.2023.11012

**Published:** 2023-05-01

**Authors:** Leah M. Santacroce, Nancy E. Avis, Alicia B. Colvin, Kristine Ruppert, Carrie Karvonen-Gutierrez, Daniel H. Solomon

**Affiliations:** 1 Division of Rheumatology, Inflammation, and Immunity, Brigham and Women’s Hospital, Boston, Massachusetts; 2 Department of Social Sciences and Health Policy, Wake Forest School of Medicine, Winston-Salem, North Carolina; 3 Department of Epidemiology, School of Public Health, University of Pittsburgh, Pittsburgh, Pennsylvania; 4 Department of Epidemiology, University of Michigan, School of Public Health, Ann Arbor

## Abstract

**Importance:**

Women often experience physiological and functional changes in their health during midlife. Identifying women who have clinically important improvements in physical health and function and evaluating the factors associated with these improvements can identify intervention targets at midlife.

**Objective:**

To identify factors associated with improvements in physical health and function among women during midlife.

**Design, Setting, and Participants:**

Participants were part of the Study of Women’s Health Across the Nation (SWAN), a diverse cohort of US women early in midlife, and followed up annually for up to 21 years between 1996 and 2017. Analyses were based on visit 8 (2004-2006) through visit 15 (2015-2017). Statistical analysis was conducted from October 2021 to March 2023.

**Exposures:**

Sociodemographic indicators, health status measures, and comorbidities measured at visit 8.

**Main Outcomes and Measures:**

The main outcome was a clinically important (≥5 points) improvement in the physical component score (PCS) of the 36-item Short-Form Health Survey between visit 8 and visit 15.

**Results:**

Of the 1807 women (at visit 8: mean [SD] age, 54.5 [2.7] years; 898 [50%] White participants) in SWAN who qualified for analysis, 265 (15%) experienced a clinically important improvement in PCS over a median of 11.1 years (IQR, 10.9-11.4 years). Factors associated with improvement in PCS included no financial strain (odds ratio [OR], 1.73; 95% CI, 1.18-2.52), no sleep disturbances (OR, 1.43; 95% CI, 1.05-1.96), no osteoarthritis (OR, 1.42; 95% CI, 1.01-1.99), and having a higher physical activity score (OR, 1.17; 95% CI, 1.00-1.37) as assessed at visit 8. Women who had a higher PCS at visit 8 (OR, 0.84; 95% CI, 0.83-0.86), who had a higher body mass index (OR, 0.95; 95% CI, 0.93-0.97), or who were taking more medications (OR, 0.93; 95% CI, 0.88-0.98) had lower odds of an improved PCS.

**Conclusions and Relevance:**

This cohort study of women in midlife suggests that approximately 15% of women experienced clinically important improvements in health and function over an 11-year period. Several potentially modifiable factors associated with improvements may inform women of variables to target for future interventions.

## Key Points

**Question:**

What factors are associated with improved physical health and function during midlife among women?

**Findings:**

In this cohort study of 1807 women, 15% experienced a clinically significant improvement in the 36-item Short-Form Health Survey physical component score (PCS). Those who had an improved PCS had statistically better sleep, less financial strain, lower body mass index, fewer medications, no osteoarthritis, a higher physical activity score, and a lower baseline PCS compared with those who did not improve.

**Meaning:**

These findings suggest that an individual’s characteristics could be useful in identifying and targeting interventions to improve and support women’s health and function during midlife.

## Teaser

This cohort study uses data from the Study of Women’s Health Across the Nation to identify factors associated with improvements in physical health and function among women during midlife.

## Introduction

Midlife is a period of important changes in health and function.^1^ Several studies have found that physical health and functional limitations commence during midlife for women and that these limitations persist throughout midlife and into late adulthood.^2,3,4^ Furthermore, evidence suggests that these limitations or decreases in physical health and function are associated with chronic conditions that develop before or during midlife.^5^ However, other work has shown the highly dynamic nature of functional status during midlife,^6^ demonstrating that some women may improve their physical health and functioning during this time.^7,8^

Good or improving physical health and functioning is an important goal and a main component of successful aging.^9^ Although successful aging is commonly studied in populations of older adults,^10,11,12^ differences in physical health and function trajectories are evident as early as midlife.^13^ Furthermore, midlife functioning has been shown to be associated with mortality and dependent living.^10^ However, as argued by Stowe and Cooney,^14^ there are many modifiable factors associated with successful aging,^9,15^ and aging is a lifelong process influenced by several static and early life experiences.^14,16^

Identifying factors associated with improvements in midlife health and function may allow for the development of targeted interventions for selected patients. To examine the prevalence of clinically important changes in physical health and function during midlife, we examined the physical component score (PCS) of the 36-item Short-Form Health Survey (SF-36) in a diverse longitudinal cohort of women in midlife. The aim of this study was to identify factors associated with clinically important improvements in PCS among women during midlife.

## Methods

In this cohort study, all participants provided written informed consent at each visit, and each Study of Women’s Health Across the Nation (SWAN) site (University of Michigan, Massachusetts General Hospital, Rush University Medical Center, University of California Davis and Kaiser Permanente, University of California at Los Angeles, Albert Einstein College of Medicine, and University of Pittsburgh) received institutional review board approval at the home institution. This report followed the Strengthening the Reporting of Observational Studies in Epidemiology (STROBE) reporting guideline for cohort studies.^17^

### Study Population and Design

This study used data from SWAN, a multisite, multiethnic, and multiracial community-based cohort study of menopause transition and midlife conducted at 7 sites in the US.^18^ Baseline visits occurred in 1996 and 1997 and enrolled 3302 participants. Entry criteria included being 42 to 52 years of age, not taking hormone therapy in the past 3 months, having an intact uterus and 1 or more ovaries, having experienced 1 or more menses in the prior 3 months, and self-identifying as one of the eligible race and ethnicity groups (Black, Chinese, Hispanic, Japanese, and White). The New Jersey site temporarily closed around the time of analytic baseline, and the women (the only Hispanic women in the study) from this site had too few follow-up visits to be included in analysis. Follow-up in SWAN began in 1998, and the most recent follow-up (visit 15) finished in 2017. The data for this analysis range from 2004 to 2017.

### Outcomes

The outcome in this analysis was a clinically important improvement in the PCS of the SF-36, a generic health status measure with 8 subscales and 2 summary scores, with one being the PCS.^19^ The PCS contains 20 items from the SF-36 and is calculated by standardizing and weighting each of the 8 domains: physical function, role limitations due to physical health, bodily pain, general health perceptions, vitality, social functioning, role limitations due to emotional problems, and mental health. The PCS is normalized from 0 to 100 to have a mean (SD) score of 50 (10), with higher scores indicating better physical health and functioning.^20^

The full SF-36 was administered at visits 6, 8, 10, 12, 13, and 15 in SWAN. We chose to examine the change in PCS over at least 10 years; therefore, we chose visit 8 (2004-2006) as the first visit and visit 15 (2015-2017) as the last visit. Ten years was chosen as a reasonable time for considering interventions; it covers the midlife period and is easily interpretable. The primary outcome was an improvement of 5 points or more^21,22^ in the PCS during the study period. The PCS at visit 8 is hereby referred to as the “baseline PCS,” with baseline referring to our analytic baseline rather than SWAN’s baseline. We required women to have complete PCS data at visits 8 and 15 and to have PCS data at 3 or more time points.

### Variables

Variables of interest included age, race and ethnicity, educational level, alcohol use, smoking status, marital status, financial strain, sleep quality, physical activity, presence of comorbid conditions, body mass index (BMI), medication use, and common symptoms and problems. All variables were taken from visit 8 except where noted. Race and ethnicity were self-reported at a screening visit prior to SWAN’s baseline. Data on educational level were collected at SWAN’s baseline and categorized into high school or less and more than high school for analysis. Alcohol use was assessed via a questionnaire and defined as less than 1 drink a week, 1 to 7 drinks a week, more than 7 drinks a week, and no drinks per week. Smoking status was defined as current smoker, past smoker, or never smoker. Marital status was defined as married; separated, divorced, or widowed; or single. Financial strain (dichotomized as “somewhat hard” or “very hard” compared with “not hard at all”) was based on self-reported difficulty paying for basics such as food, housing, medical care, and heating. Participants were considered to have sleep disturbance if they self-reported trouble falling asleep, waking up several times, or waking up too early 3 to 4 times per week for the past 2 weeks.^23^ Physical activity was measured by a 4-question sports index, part of the Kaiser Physical Activity Score that ranged from 1 to 5 points, with higher numbers indicating higher levels of activity through sports.^24^ Questions asked whether the women played sports or exercised, sweated from exertion, what sports or exercise they did, and how frequently they played sports or exercised. This score was not available at visit 8, so values from visit 6 were used.

The presence of comorbid conditions at visit 8 was based on self-report of ever having the following conditions: hypertension, hyperlipidemia, thyroid disease, osteoarthritis, osteoporosis, diabetes, cardiovascular disease (myocardial infarction, stroke, or angina), and cancer. Each comorbidity was considered as a binary variable (yes or no) as well as a total count of the number of all comorbidities. Body mass index was calculated as weight in kilograms divided by height in meters squared by trained technicians. A full prescription medication inventory was obtained; the number of prescription medications reported was tabulated for each participant and considered as a continuous variable. Several common problems or symptoms were evaluated, and a binary variable was created for each one if a woman experienced it 6 to 8 days or more in the 2 weeks preceding visit 8. The common problems and symptoms included back aches or pains, vaginal dryness, feeling “blue” or depressed, dizzy spells, forgetfulness, frequent mood changes, heart pounding or racing, feeling fearful for no reason, headaches, and breast pain or tenderness. If values were missing at visit 8, we imputed using the most proximal nonmissing values from previous visits. Participants with values still missing for key variables were excluded from final analysis.

### Statistical Analysis

Statistical analysis was conducted from October 2021 to March 2023. Descriptive analyses for our cohort were calculated. The primary analysis used logistic regression models to evaluate associations between the aforementioned covariates and clinically important PCS improvement. Model building first included evaluation of the association between each individual covariate and the outcome, with each model adjusted for baseline PCS. Then, all variables with *P* < .10 were added to a multivariable model; variables with *P* ≥ .10 were dropped to obtain the final adjusted logistic model. Age was kept in the model despite lack of statistical significance. We created models with or without adjusting for baseline PCS, and based on the results of these models, we ran several post hoc models, one set stratified by tertiles of baseline PCS and another with several interaction terms. An interaction term with each variable and the baseline PCS was evaluated, and the terms significant at an α level of .05 were kept in the model. To aid in interpreting interaction terms, we generated several odds ratios (ORs) at different PCS starting points below and at the national median (50).

To evaluate the association of changes in the values of variables over time (identified post hoc) with physical health and function, we plotted BMI, number of medications, physical activity score, and sleep disturbance data over study visits. A large improvement in PCS was observed between visit 8 and visit 10. Thus, we also calculated summary statistics for several variables at visit 8 and visit 10 and their changes in a post hoc descriptive analysis.

Significance testing was performed using 2-sided tests with *P* < .05 considered statistically significant. Statistical analyses were conducted using SAS, version 9.4 (SAS Institute Inc) and R, version 4.1.12 (R Group for Statistical Computing).

## Results

**Figure 1.**
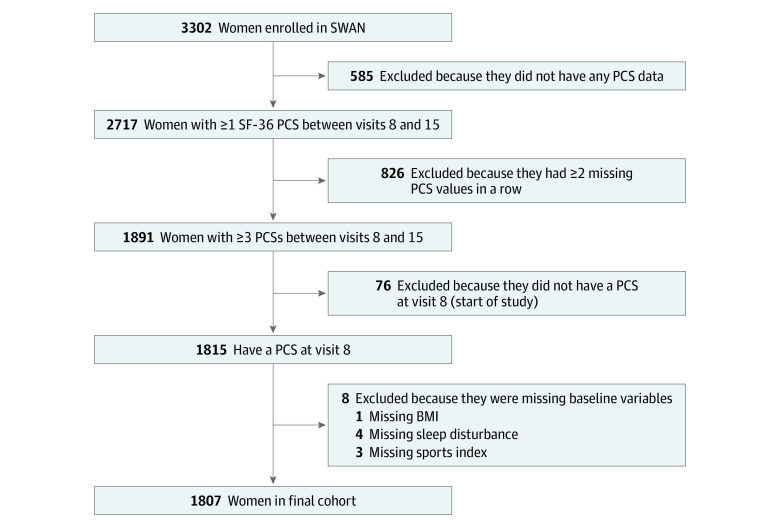
Derivation of Analytic Sample The inclusion and exclusion criteria and the number of women excluded at each step to arrive at the analytic sample are shown. BMI indicates body mass index; PCS, physical component score; SF-36, 36-item Short-Form Health Survey; and SWAN, Study of Women’s Health Across the Nation.

The analytic sample included 1807 women (at visit 8: mean [SD] age, 54.5 [2.7] years; 898 [50%] White participants; and mean [SD] BMI, 29 [7]) (Figure 1; Table 1). Women in the SWAN cohort who were excluded from these analyses were compared with the analytic cohort (eTable 1 in Supplement 1). The percentages of certain comorbidities and symptoms were higher for those who were excluded. Across the included sample, 265 women (15%) had a clinically important (≥5 point) PCS improvement over a median of 11.1 years (IQR, 10.9-11.4 years) (Table 1). Nearly one-quarter of women (433 [24%]) reported financial strain. The data are also stratified by those who did and those who did not have a clinically important PCS improvement. The proportions of certain common problems, such as back aches and pains (40% [106 of 265] vs 22% [338 of 1542]) and stiffness (55% [146 of 265] vs 36% [558 of 1542]), as well as certain comorbidities, including osteoarthritis (50% [133 of 265] vs 40% [615 of 1542]) and osteoporosis (17% [46 of 265] vs 8% [130 of 1542]), were higher at baseline among those whose PCS improved.

**Table 1.** Characteristics of Study Cohort at Visit 8 in Study of Women’s Health Across the Nation

Characteristic at visit 8	Patients, No. (%)		
	Full cohort (N = 1807)	With PCS improvement (n = 265)	Without PCS improvement (n = 1542)
Age, mean (SD), y	54.5 (2.7)	54.4 (2.7)	54.5 (2.7)
SF-36 PCS, mean (SD)	51 (9)	42 (9)	52 (8)
BMI, mean (SD)	29 (7)	29 (8)	29 (7)
No. of medications, median (IQR)	2 (0-3)	2 (1-4)	2 (1-4)
No. of comorbidities, median (IQR)	2 (1-3)	2 (1-3)	2 (1-3)
Physical activity score (range, 1-5), mean (SD)	2.8 (1.0)	2.6 (1.0)	2.8 (1.0)
Race and ethnicity			
Black	493 (27)	67 (25)	426 (28)
Chinese	193 (11)	38 (14)	155 (10)
Japanese	223 (12)	35 (13)	188 (12)
White	898 (50)	125 (47)	773 (50)
Less than college education	911 (50)	132 (50)	779 (51)
Alcohol use, No./total No. (%)			
<1 Drink/wk	330/1310 (25)	43/178 (24)	287/1132 (25)
1-7 Drinks/wk	244/1310 (19)	30/178 (17)	214/1132 (19)
>7 Drinks/wk	115/1310 (9)	13/178 (7)	102/1132 (9)
None	621/1310 (47)	92/178 (52)	529/1132 (47)
Smoking status			
Current	177 (10)	22 (8)	155 (10)
Never	1097 (61)	167 (63)	930 (60)
Past only	532 (29)	76 (29)	456 (30)
Marital status			
Married	1193 (66)	168 (64)	1025 (66)
Separated, divorced, or widowed	401 (22)	59 (22)	342 (22)
Single	212 (12)	37 (14)	175 (11)
Menopause status			
Early perimenopausal	327 (18)	46 (17)	281 (18)
Late perimenopausal	161 (9)	22 (8)	139 (9)
Postmenopausal, surgical	87 (5)	16 (6)	71 (5)
Postmenopausal, natural	1092 (60)	163 (62)	929 (60)
Premenopausal	24 (1)	2 (1)	22 (1)
Unknown, hormone therapy	74 (4)	11 (4)	63 (4)
Unknown, status after hysterectomy	41 (2)	5 (2)	36 (2)
Financial strain^a^	433 (24)	69 (26)	364 (24)
Sleeping disturbance^b^	802 (44)	127 (48)	675 (44)
Diabetes	212 (12)	40 (15)	172 (11)
Hyperlipidemia	801 (44)	132 (50)	669 (43)
Hypertension	690 (38)	109 (41)	581 (38)
CVD	106 (6)	22 (8)	84 (5)
Osteoarthritis	748 (41)	133 (50)	615 (40)
Osteoporosis	176 (10)	46 (17)	130 (8)
Thyroid disease	348 (19)	55 (21)	293 (19)
Cancer	96 (5)	13 (5)	83 (5)
Depressive symptoms^c^	275 (15)	40 (15)	235 (15)
Common problems or symptoms			
Back aches or pain	444 (25)	106 (40)	338 (22)
Cold sweats	64 (4)	13 (5)	51 (3)
Night sweats	252 (14)	37 (14)	215 (14)
Vaginal dryness	271 (15)	45 (17)	226 (15)
Feeling “blue”	205 (11)	33 (12)	172 (11)
Dizzy spells	41 (2)	11 (4)	30 (12)
Forgetfulness	380 (21)	73 (28)	307 (20)
Mood changes	137 (8)	26 (10)	111 (7)
Heart racing or pounding	87 (5)	18 (7)	69 (5)
Fearful for no reason	38 (2)	5 (2)	33 (2)
Headaches	150 (8)	29 (11)	121 (8)
Breast pain	65 (4)	11 (4)	54 (4)
Hot flashes	469 (26)	74 (28)	395 (26)
Stiffness	704 (39)	146 (55)	558 (36)
Irritability	205 (11)	29 (11)	176 (11)
Nervousness	219 (12)	30 (11)	189 (12)

Abbreviations: BMI, body mass index (calculated as weight in kilograms divided by height in meters squared); CVD, cardiovascular disease; PCS, physical component score; SF-36, 36-item Short-Form Health Survey.

^a^ Financial strain was defined as having a “somewhat hard” or “very hard” time paying for basics such as food, housing, medical care, and heating.

^b^ Sleeping disturbance was defined as at least 3 or more nights per week of difficulty initiating sleep, difficulty remaining asleep, or early morning awakenings.

^c^ Depressive symptoms defined as a Center for Epidemiologic Studies-Depression score of 16 or more.

**Figure 2.**
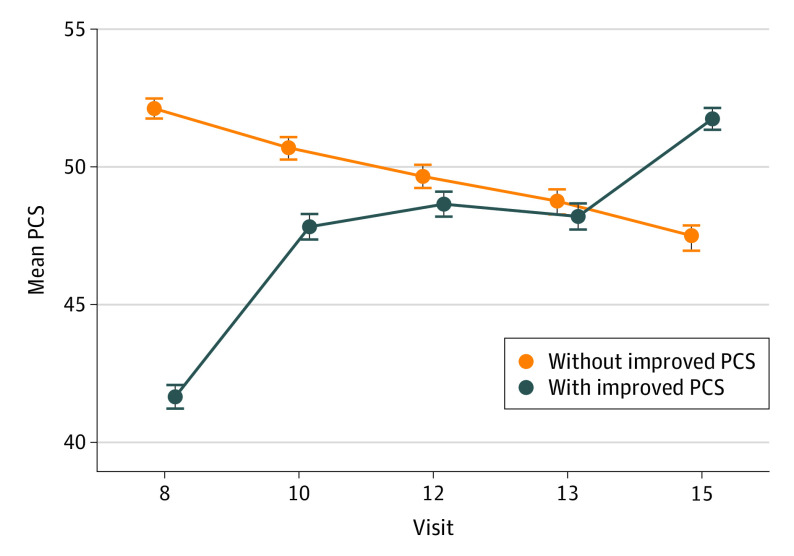
Mean Physical Component Score (PCS) at Each Time Point Stratified by Women With Improved PCS vs Women Without Improved PCS The mean (SD) for the 36-item Short-Form Health Survey PCS for women whose PCS improved by at least 5 points (n = 265) and for women whose PCS did not improve (n = 1542) is shown. Error bars indicate the SD.

The mean PCS at each visit stratified by improvement status is illustrated in Figure 2. The women who had a clinically important PCS improvement had a mean (SD) increase of 10.3 (5.2) points from visit 8 to visit 15, and the PCS improvement occurred mostly between visit 8 and visit 10. Of the 265 women with an improved PCS, 215 (81%) started below the national PCS mean of 50, and 106 (40%) started 1 SD below the national mean (40). Because many improvements in PCS occurred between visit 8 and visit 10, we subsequently examined changes in selected variables from visit 8 to visit 10 among all women (eTable 2 in Supplement 1). Among the women with an improvement in PCS, 17 (6%) had a reduction in back aches and pains, 20 (8%) had a reduction in stiffness, and 13 (5%) had a reduction in forgetfulness. Women without an improvement in PCS had both increases and decreases in symptoms, with smaller percentage changes.

**Figure 3.**
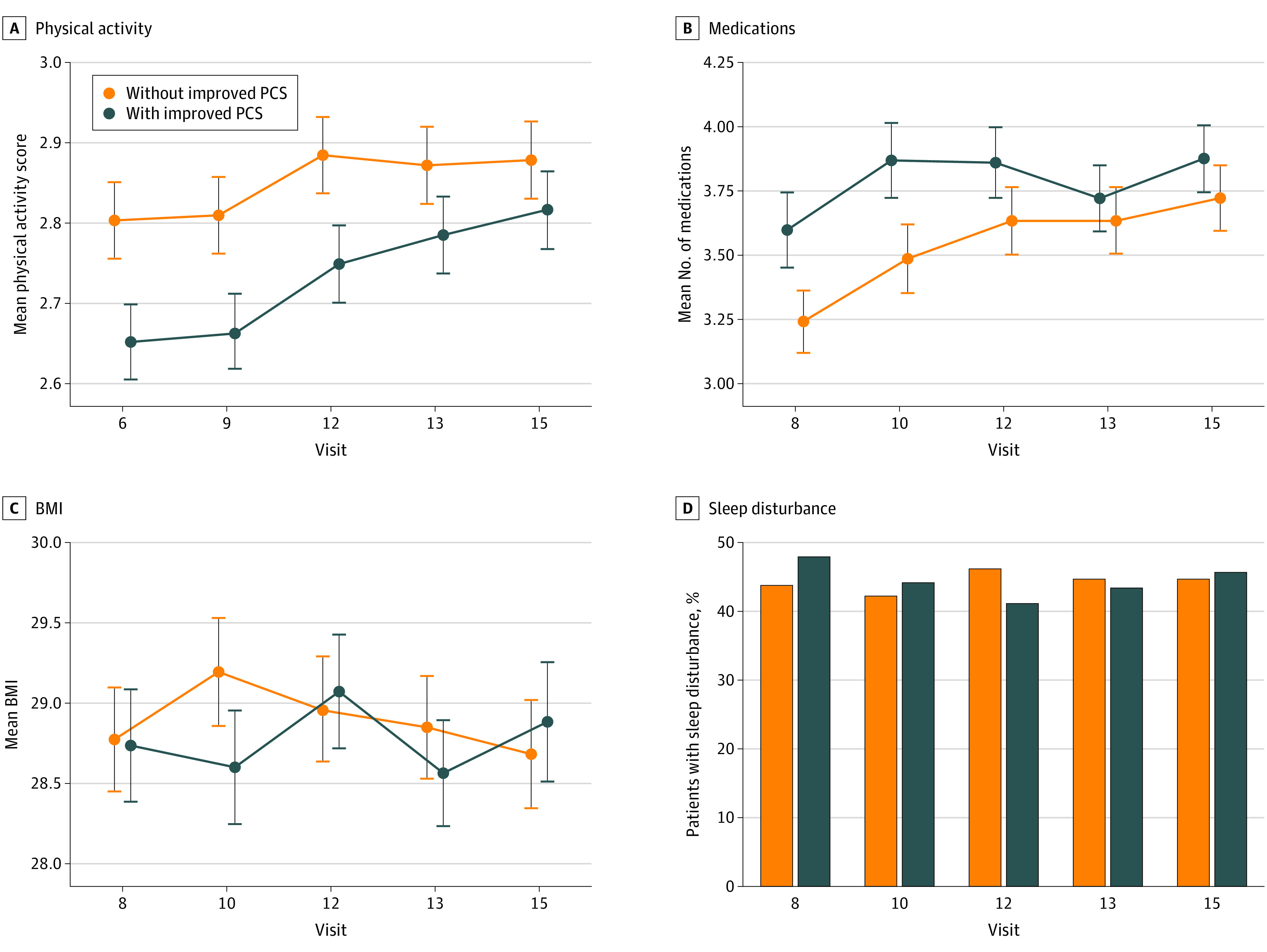
Physical Activity, Medications, Body Mass Index (BMI), and Sleep Disturbance at Each Study Time Point Stratified by Women Whose Physical Component Score (PCS) Improved vs Those Whose PCS Did Not Improve This figure illustrates how 4 covariates change over time for women whose PCS improved by at least 5 points vs those whose PCS did not improve. Continuous variables (BMI [calculated as weight in kilograms divided by height in meters squared], number of medications, and physical activity) are represented as mean (SD) values, while the binary variable (sleep disturbance) is represented by percentages. Error bars indicate the SD.

To further examine the changes in selected variables over time, we graphed these changes for those with an improved PCS and for those without an improved PCS. As shown in Figure 3, women who had clinically important improvements in PCS had greater increases in physical activity across follow-up compared with women who did not have an improved PCS (0.16 vs 0.07 points), with the biggest increase occurring between visit 9 and visit 12. Those with an improved PCS started with a higher number of medications (mean, 3.6) that remained constant over time, while those who did not have an improved PCS showed an increase in medications (ending with a mean of 3.7) to the level of those with an improvement. Women in both groups had a fluctuating BMI over time. Women with an improved PCS started with a slightly higher percentage of sleep disturbance (48% vs 44%) but, by the end of follow-up, had a similar percentage of sleep disturbance as women who did not have an improved PCS (46% vs 45%). These covariates are also graphed and stratified by baseline PCS in the eFigure in Supplement 1, which shows that women who are in the lowest tertile of PCS at baseline have less physical activity, more medications, higher BMI, and more sleep disturbances over time.

Table 2 shows results from the primary multivariable logistic regression models. Among the entire cohort, the statistically significant factors associated with clinically important PCS improvements were a lower BMI (OR, 0.95; 95% CI, 0.93-0.97), no financial strain (OR, 1.73; 95% CI, 1.18-2.52), no sleep disturbance (OR, 1.43; 95% CI, 1.05-1.96), fewer medications (OR, 0.93; 95% CI, 0.88-0.99), no osteoarthritis (OR, 1.42; 95% CI, 1.01-1.99), a lower baseline PCS (OR, 0.84; 95% CI, 0.83-0.86), and having a higher physical activity score (OR, 1.17; 95% CI, 1.00-1.37). Also shown are the results of 3 models stratified by tertile of baseline PCS. Tertile 1, which includes women who started with a lower PCS (<49.4), shows similar results to the entire cohort, but the higher-tertile models lack statistical significance, possibly due to the much smaller number of women with the outcome in the higher tertiles, further suggesting that most of the women with an improved PCS started with a lower PCS. For comparison purposes, we added the full cohort model without baseline PCS in eTable 3 in Supplement 1. The direction and significance of several variables changed with the addition of the baseline PCS.

**Table 2.** Results of Logistic Regression Models: Odds of Improvement of 5 Points or More in PCS

Characteristic at visit 8^a^	Odds ratio (95% CI)			
	Model with full cohort, primary model	Tertile 1 (PCS <49.4)	Tertile 2 (PCS 49.4-55.7)	Tertile 3 (PCS >55.7)
No.	1807	602	602	603
Age	0.96 (0.91-1.02)	0.94 (0.88-1.00)	0.98 (0.97-1.09)	1.07 (0.80-1.41)
No sleep disturbance	1.43 (1.05-1.96)	1.58 (1.10-2.29)	1.04 (0.56-1.95)	3.35 (0.60-67.1)
No financial strain	1.73 (1.18-2.52)	1.56 (1.04-2.36)	2.24 (0.92-6.74)	0.42 (0.09-3.05)
No osteoarthritis	1.42 (1.01-1.99)	1.51 (1.02-2.25)	1.28 (0.66-2.61)	2.57 (0.42-50.6)
Physical activity	1.17 (1.00-1.37)	1.34 (1.10-1.63)	1.01 (0.74-1.36)	1.53 (0.71-3.58)
Body mass index	0.95 (0.93-0.97)	0.96 (0.94-0.98)	0.93 (0.87-0.98)	0.90 (0.74-1.06)
No. of medications	0.93 (0.88-0.99)	0.95 (0.90-1.01)	0.92 (0.77-1.06)	0.98 (0.60-1.33)
Baseline PCS	0.84 (0.83-0.86)	0.91 (0.88-0.93)	0.85 (0.72-1.00)	0.46 (0.19-0.84)
No. with outcome (change of ≥5 points in PCS), No. (%)	265 (15)	209 (35)	48 (8)	8 (1)

Abbreviation: PCS, physical component score.

^a^ Variables in the models were selected based on logistic regression adjusting for baseline PCS at visit 8. Age was forced into all models despite lack of statistical significance.

Due to the findings of the association of baseline PCS with change in physical health and function in the models, we present the primary model with 2 additional interaction terms: PCS with osteoarthritis and PCS with physical activity. Both interaction terms were statistically significant. eTable 4 in Supplement 1 shows the *P* values and selected ORs of the model. The results are similar to the primary model, with no sleep disturbance (OR, 1.48; 95% CI, 1.08-2.02), no financial strain (OR 1.59; 95% CI 1.09-2.31), BMI (OR 0.95, 95% CI 0.93-0.97), and number of medications (OR 0.94, 95% CI 0.89-0.99) associated with improved PCS (Table 2). Osteoarthritis, physical activity, and their interaction terms were also statistically significant. eTable 5 in Supplement 1 shows several ORs generated for different levels of PCS (40, 45, and 50). The results show that the association of both osteoarthritis and physical activity with an improved PCS over time are stronger and more significant among women with lower baseline levels of PCS. The OR for no osteoarthritis at a baseline PCS of 40 was 2.25 (95% CI, 1.49-3.39), while at a PCS of 50 it was 1.06 (95% CI, 0.74-1.51). The OR for physical activity at a baseline PCS of 40 was 1.36 (95% CI, 1.12-1.65), while at a PCS of 50 it was 1.08 (95% CI, 0.91-1.28).

## Discussion

We studied patterns of self-reported physical health and function over time to identify women who had clinically important improvements during midlife and to identify correlates of improvements. Approximately 15% of women in midlife demonstrated clinically significant improvements in physical health and function over an 11-year period. Having a lower baseline PCS was associated with having clinically important improvements in health and function, suggesting that women who start with a lower PCS have room to improve. This finding supports previous research demonstrating that midlife is a period of highly dynamic patterns in functioning and that improvements can occur even among those with previously poor functioning.^8^ Our findings demonstrate that women whose PCS improved had many symptoms at the beginning of our study (visit 8). By the next study visit, however, many women had experienced resolution of these symptoms, suggesting that symptom resolution may be an important feature associated with improvements in health and function.

Our findings identified several important factors associated with improved health and function during midlife, including less financial strain, lower BMI, fewer medications, less likely to have a sleep disturbance, higher level of physical activity, and no osteoarthritis. Our findings show that the PCS at the start of follow-up is associated with the outcome as well as the covariates. The results of the models stratified by baseline PCS and the models with interaction terms suggest that many of the factors that we identified are significantly associated with clinically important improvements among women who start with a lower PCS, but the associations were not significant among those with a high baseline PCS. Several variables appeared to be associated with improvement in multivariable models without a baseline PCS but either lost significance or changed direction with the addition of baseline PCS, likely owing to the interactions between PCS and several variables.

Prior studies have focused on positive aging and physical health and function. These investigations have found that a higher BMI and medical conditions are associated with a lower PCS, which is consistent with our findings.^2,7,25^ Studies among elderly populations have shown that socioeconomic status was significantly associated with positive aging,^11^ which is consistent with our findings that having no financial strain was associated with improved physical health and functioning. It has also been found that good sleep supports the maintenance of physical health and functioning, which was replicated in our findings.^26^ Our study adds to the literature by identifying factors associated with clinically meaningful physical health and function improvements among women in midlife and by confirming factors associated with general positive aging in midlife and in older individuals.

Identifying patterns of change in health and function during midlife is critical to optimize healthy aging trajectories. The low baseline PCS for many women gives credence to evidence demonstrating that midlife is a time of important changes in health and function. Furthermore, the findings of a moderate prevalence of improvement suggests that health and function are highly amenable to change during this life stage. Thus, knowledge regarding factors associated with improvement can be leveraged as potential targets for intervention, and women with poor health and function who do not exhibit these factors may be identified for greater support across midlife.

### Limitations and Strengths

Several limitations of these analyses should be noted. Because we required women to have several visits at least 10 years apart, some women in the cohort had dropped out of SWAN prior to visit 15. This could create a bias against women who were not healthy enough to remain in the study. The question on comorbidities assessed only whether the comorbidity had ever occurred, making it difficult to identify why some women had a lower PCS at baseline. Because this study took place at only 7 sites across the US, there is limited ability to generalize to women in geographic areas not represented. The findings also may not be generalizable to Hispanic populations because they were excluded from this analysis.

Despite these limitations, our analyses have several strengths. A main strength of this study is that it provides a clinically meaningful and interpretable model for women who improve their physical health and function over an 11-year period. In addition, the SWAN cohort was racially, ethnically, and geographically diverse. The SWAN questionnaire collected a broad range of variables that we tested in regression models. This study captured health-related quality-of-life information at 6 different time points.

## Conclusions

In this cohort study of women during midlife, we identified several factors associated with improvements in physical health and functioning, including baseline physical health, lack of sleep disturbance, lack of financial strain, lack of osteoarthritis, higher physical activity, lower BMI, and lower number of medications. Midlife is an important time to study and intervene on physical health and function since there are likely modifiable patterns and risk factors. Identifying these risk factors during this window of opportunity is the first step toward developing interventions. Successful aging is an important goal, but it is a broad and multifaceted concept, and thus it is difficult to investigate and compare results across multiple studies. Health status fluctuates over time during midlife, and some women improve while others do not; this study suggests there is a lot to learn about what events are associated with changing health. The results of this study are likely to help us develop strategies for interventions to support physical health improvements in midlife.

## Notes

Supplement 1. **eTable 1.** Characteristics of Included Cohort and Excluded Cohort at SWAN Baseline **eTable 2.** Change in Select Covariates From Visit 8 to 10 **eTable 3.** Logistic Regression Model Without Baseline PCS **eTable 4.** Primary Logistic Model Predicting Odds of PCS Improvement With Baseline PCS Interaction Terms **eTable 5.** Interaction Odds Ratios for PCS Improvement at Different Levels of Baseline PCS for Osteoarthritis and Physical Activity **eFigure.** Select Covariates Over Time Stratified by Different Tertiles of Baseline PCS

Supplement 2.
Data Sharing Statement
