# Supplementary material for: Physical and Behavioral Factors Associated With Improvement in Physical Health and Function Among US Women During Midlife
Source: JAMA Netw Open. 2023 May 1;6(5):e2311012. doi: 10.1001/jamanetworkopen.2023.11012 (PMC10152304; doi:10.1001/jamanetworkopen.2023.11012)
Supplement: Supplement 1. — eTable 1. Characteristics of Included Cohort and Excluded Cohort at SWAN Baseline eTable 2. Change in Select Covariates From Visit 8 to 10 eTable 3. Logistic Regression Model Without Baseline PCS eTable 4. Primary Logistic Model Predicting Odds of PCS Improvement With Baseline PCS Interaction Terms eTable 5. Interaction Odds Ratios for PCS Improvement at Different Levels of Baseline PCS for Osteoarthritis and Physical Activity eFigure. Select Covariates Over Time Stratified by Different Tertiles of Baseline PCS [file jamanetwopen-e2311012-s001.pdf]

Supplementary Online Content

Santacroce LM, Avis NE, Colvin AB, Ruppert K, Karvonen-Gutierrez C, Solomon DH. Physical and behavioral factors associated with improvement in physical health and function among US women during midlife. *JAMA Netw Open.* 2023;6(5):e2311012. doi:10.1001/jamanetworkopen.2023.11012

eTable 1. Characteristics of Included Cohort and Excluded Cohort at SWAN Baseline
eTable 2. Change in Select Covariates From Visit 8 to 10
eTable 3. Logistic Regression Model Without Baseline PCS
eTable 4. Primary Logistic Model Predicting Odds of PCS Improvement With Baseline PCS Interaction Terms
eTable 5. Interaction Odds Ratios for PCS Improvement at Different Levels of Baseline PCS for Osteoarthritis and Physical Activity
eFigure. Select Covariates Over Time Stratified by Different Tertiles of Baseline PCS

This supplementary material has been provided by the authors to give readers additional information about their work.

© 2023 Santacroce LM et al. *JAMA Network Open.*

eTable 1. Characteristics of Included Cohort and Excluded Cohort at SWAN Baseline

Characteristic at SWAN Baseline (Visit 0)	Included Cohort (N = 1,807)	Excluded Cohort (N = 1,495)
	Patients, No. (%)	
Age, mean (SD), y	46.42 (2.66)	46.23 (2.72)
Body Mass Index (kg/m2), mean (SD)	28 (7)	29 (7)
Number of Medications, median (Q1, Q3)	0.0 (0.0, 2.0)	0.0 (0.0, 2.0)
Number of Comorbidities, median (Q1, Q3)	0.0 (0.0, 1.0)	1.0 (0.0, 1.0)
Physical Activity Score (range 1-5), mean (SD)	2.72 (1.03)	2.44 (1.00)
Race/Ethnicity		
Black	493 (27)	441 (29)
Chinese	193 (11)	57 (3.8)
Japanese	223 (12)	58 (3.9)
White	898 (50)	653 (44)
Hispanic	0 (0)	286 (19)
Less than College Education	911 (50)	959 (64)
Alcohol Use, No./Total No. (%)		
<1/week	153/1723 (8.9)	173/1410 (12)
>7/week 1-7/week	445/1723 (26)	368/1410 (26)
>7/week	272/1723 (16)	169/1410 (12)
None	853/1723 (50)	700/1410 (50)
Smoking Status		
Current	231 (13)	349 (23)
Never Smoked	1,107 (61)	783 (52)
Past Only	467 (26)	363 (24)
Menopause Status		
Early Perimenopausal	801 (44)	700 (47)
Premenopausal	997 (55)	773 (52)
Unknown, hormone therapy	3 (0.2)	3 (0.2)
Financial Strain[A]		
Sleeping Disturbance[B]	529 (29)	492 (33)
Diabetes	79 (4.4)	84 (5.6)
Hyperlipidemia	327 (18)	287 (19)
Hypertension	313 (17)	329 (22)
CVD	33 (1.8)	62 (4.1)
Osteoarthritis	282 (16)	278 (19)
Osteoporosis	25 (1.4)	15 (1.0)
Thyroid Disease	190 (11)	150 (10)
Cancer	36 (2.0)	29 (2.0)
Depressive Symptoms[C]	375 (21)	429 (29)
Common Problems/Symptoms		
Cold sweats	28 (1.6)	54 (3.7)
Night sweats	97 (5.4)	121 (8.1)
Vaginal dryness	86 (4.8)	89 (6.0)

© 2023 Santacroce LM et al. *JAMA Network Open.*

Feeling blue	203 (11)	205 (14)
Dizzy spells	26 (1.4)	58 (3.9)
Forgetfulness	288 (16)	312 (21)
Mood changes	187 (10)	192 (13)
Heart racing/pounding	85 (4.7)	104 (7.0)
Fearful for no reason	61 (3.4)	87 (5.9)
Headaches	181 (10)	206 (14)
Hot flashes	125 (6.9)	129 (8.7)
Stiffness	500 (28)	449 (30)

Notes: Abbreviations: CVD, cardiovascular disease. A. Financial strain was defined as having a "somewhat hard" or "very hard" time paying for basics such as food, housing, medical care, and heating. B. Sleeping disturbance was defined as at least 3 or more nights per week of difficulty initiating sleep, difficulty remaining asleep, or early morning awakenings. C. Depressive symptoms defined as CES-D (Center for Epidemiologic Studies-Depression) ≥16.

© 2023 Santacroce LM et al. *JAMA Network Open.*

eTable 2. Change in Select Covariates From Visit 8 to 10

	PCS Improvers (N = 265)			PCS Non-Improvers (N = 1,542)		
Characteristic	Visit 8	Visit 10	Change	Visit 8	Visit 10	Change
	Patients, No. (%)					
Age, mean (SD)	54.4 (2.7)	56.4 (2.7)	2.0 (0.2)	54.5 (2.7)	56.5 (2.7)	2.0 (0.2)
Physical Component Score, mean (SD)	41.7 (9.3)	47.8 (10.0)	6.2 (8.6)	52.1 (7.9)	50.7 (8.8)	-1.47 (6.7)
Body Mass Index (kg/m2), mean (SD)	28.9 (8.1)	28.7 (7.8)	-0.18 (2.2)	28.7 (7.3)	28.9 (7.3)	0.24 (2.0)
Number of Medications, median (Q3, Q3)	2 (1, 4)	2 (1, 5)	0 (-1, 1)	1 (0, 3)	2 (0, 4)	0 (0, 1)
Physical Activity Score, mean (SD)[A]	2.6 (1.0)	2.7 (1.0)	0.02 (0.8)	2.7 (1.0)	2.8 (1.0)	0.01 (0.8)
Financial Strain	69 (26)	68 (26)	-1 (0.4)	364 (24)	368 (24)	4 (0.2)
Sleep Disturbance	127 (48)	117 (44)	-10 (4)	675 (44)	651 (42)	-24 (2)
Common Symptoms/Problems						
Back aches/pain	106 (40)	89 (35)	-17 (6)	338 (22)	368 (24)	30 (2)
Cold sweats	13 (5)	11 (4)	-2 (1)	51 (3.3)	49 (3.2)	-2 (0.1)
Night sweats	37 (14)	29 (11)	-8 (3)	215 (14)	200 (13)	-15 (1)
Vaginal dryness	45 (17)	53 (20)	8 (3)	226 (15)	260 (17)	34 (2)
Feeling blue	33 (12)	32 (12)	-1 (0.4)	172 (11)	155 (10)	-17 (1)
Dizzy spells	11 (4.2)	10 (3.9)	-1 (0.4)	30 (2)	34 (2)	4 (0.2)
Forgetfulness	73 (28)	60 (23)	-13 (5)	307 (20)	309 (20)	2 (0.1)
Mood changes	26 (10)	20 (7)	-6 (2)	111 (7)	101 (6)	-10 (1)
Heart racing/pounding	18 (7)	17 (7)	-1 (0.4)	69 (4)	61 (4)	-8 (0.5)
Fearful for no reason	5 (2)	8 (3)	3 (1)	33 (2)	42 (3)	9 (1)
Headaches	29 (11)	23 (9)	-6 (1)	121 (8)	115 (7)	-6 (1)
Breast pain	11 (4.2)	9 (3.5)	-2 (0.7)	54 (4)	49 (3)	-5 (1)
Hot flashes	74 (28)	66 (25)	-8 (3)	395 (26)	336 (27)	-59 (4)
Stiffness	146 (55)	126 (49)	-20 (8)	558 (36)	587 (38)	29 (2)
Irritability	29 (11)	20 (8)	-9 (3)	176 (11)	152 (10)	-24 (1)
Nervousness	30 (11)	31 (12)	1 (0.4)	189 (12)	165 (11)	-24 (1)

Notes: A. Physical activity available at Visit 6 and 9, not 8 and 10. Comorbidities not included in this table because they can only increase, not decrease due to the fact they are asked as an “ever” question.

© 2023 Santacroce LM et al. *JAMA Network Open.*

eTable 3. Logistic Regression Model Without Baseline PCS

Characteristic at Visit 8	Model without baseline PCS
	OR (95% CI)
Age	0.98 (0.93-1.03)
No sleep disturbance	0.92 (0.70-1.20)
No financial strain	0.99 (0.73-1.36)
No osteoarthritis	0.70 (0.53-0.93)
Physical Activity	0.88 (0.77-1.01)
BMI	0.99 (0.97-1.01)
Number of medications	1.05 (1.00-1.10)

Notes: Abbreviations: PCS, Physical Component Score; BMI, Body Mass Index.

© 2023 Santacroce LM et al. *JAMA Network Open.*

eTable 4. Primary Logistic Model Predicting Odds of PCS Improvement With Baseline PCS Interaction Terms

Characteristic at Visit 8	Model with PCS and interaction terms	
	OR (95% CI)	P-value
Age	0.96 (0.90-1.01)	0.13
No sleep disturbance	1.48 (1.08-2.02)	0.016
No financial strain	1.59 (1.09-2.31)	0.016
No osteoarthritis	*	<0.001
Physical Activity	*	0.003
BMI	0.95 (0.93-0.97)	<0.001
Number of medications	0.94 (0.89-0.99)	0.044
Baseline PCS	*	<0.001
Baseline PCS * No osteoarthritis	*	<0.001
Baseline PCS * Physical Activity	*	0.008

Notes: Abbreviations: BMI, Body Mass Index; PCS, Physical Component Score. Single odds ratio values not available for terms with interaction. Interaction terms with baseline PCS and all other variables evaluated in the primary model, osteoarthritis and physical activity were statistically significant and remained in model.

© 2023 Santacroce LM et al. *JAMA Network Open.*

eTable 5. Interaction Odds Ratios for PCS Improvement at Different Levels of Baseline PCS for Osteoarthritis and Physical Activity

Variable and Level	Odds Ratio (95% Confidence Interval)
No Osteoarthritis vs Osteoarthritis	
PCS of 40 at Visit 8	2.25 (1.49-3.39)
PCS of 45 at Visit 8	1.54 (1.10-2.15)
PCS of 50 at Visit 8	1.06 (0.74-1.51)
1 Unit Increase in Physical Activity	
PCS of 40 at Visit 8	1.36 (1.12-1.65)
PCS of 45 at Visit 8	1.21 (1.03-1.42)
PCS of 50 at Visit 8	1.08 (0.91-1.28)

Notes: Abbreviations: PCS, Physical Component Score. Odds Ratios generated for several different levels of baseline PCS for both osteoarthritis and physical activity due to the interaction terms. PCS levels set at 40, 45, and 50.

© 2023 Santacroce LM et al. *JAMA Network Open.*

eFigure. Select Covariates Over Time Stratified by Different Tertiles of Baseline PCS

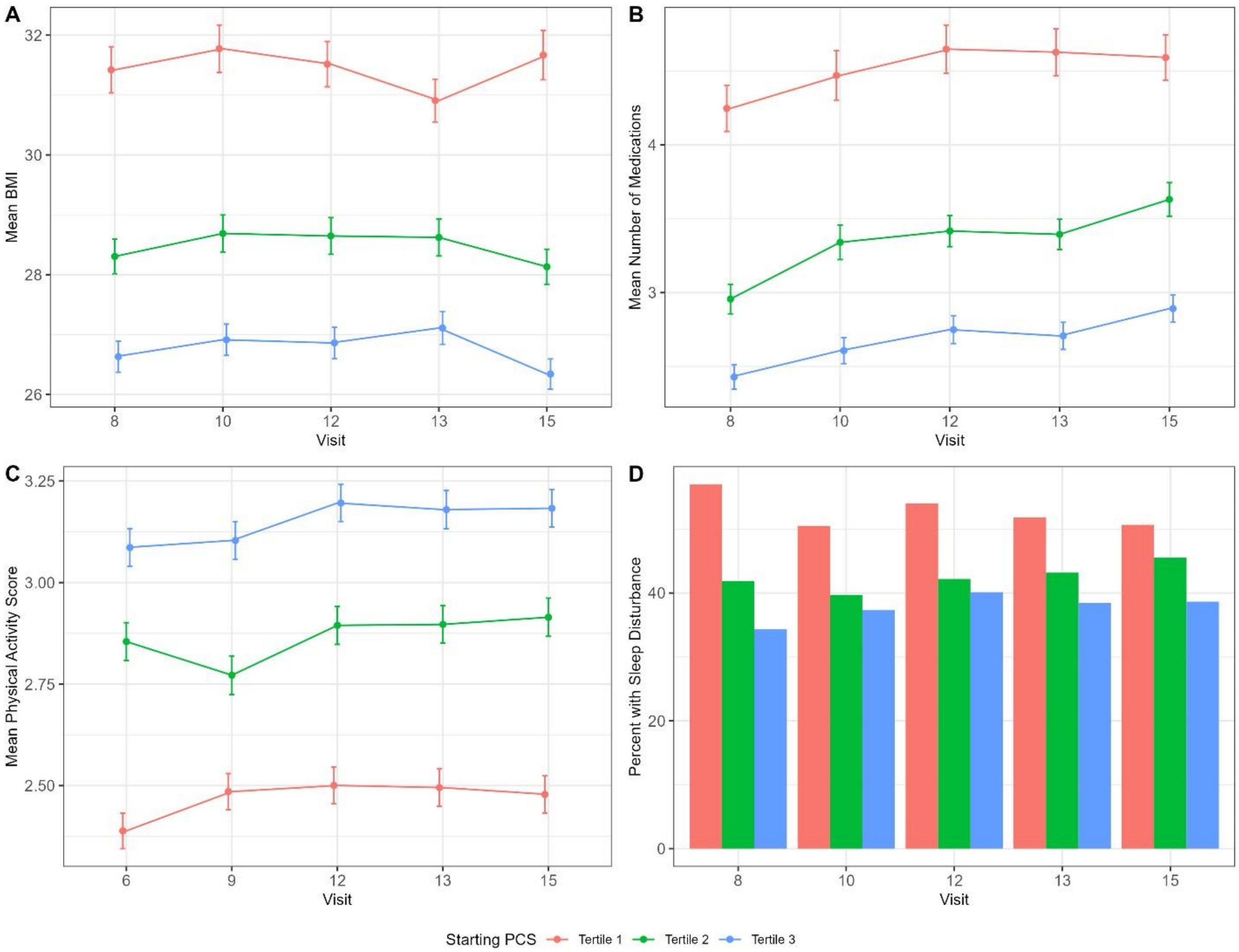

Legend: This figure demonstrates how four covariates change over time stratified by different tertiles of PCS at visit 8. The variables graphed are Physical Activity Score, Number of Medications Body Mass Index (BMI), and Sleep Disturbance. Continuous variables (BMI, number of medications, physical activity) are represented by the mean and standard deviation while binary variables (Sleep disturbance) are represented by percentages.

© 2023 Santacroce LM et al. *JAMA Network Open.*
