# Supplementary material for: Physical and Behavioral Factors Associated With Improvement in Physical Health and Function Among US Women During Midlife
Source: JAMA Netw Open. 2023 May 1;6(5):e2311012. doi: 10.1001/jamanetworkopen.2023.11012 (PMC10152304; doi:10.1001/jamanetworkopen.2023.11012)
Supplement: Supplement 2. — Data Sharing Statement [file jamanetwopen-e2311012-s002.pdf]

Data Sharing Statement

Santacroce. Physical and Behavioral Factors Associated With Improvement in Physical Health and Function Among US Women During Midlife. *JAMA Netw Open*. Published May 01, 2023. doi:10.1001/jamanetworkopen.2023.11012

Data
Data available: No

Additional Information
Explanation for why data not available: This research is part of SWAN, the Study of Women's Health Across the Nation, which is an NIH funded study that allows outside investigators to access the data through an approval process.
